# Comparing multiple competing interventions in the absence of randomized trials using clinical risk-benefit analysis

**DOI:** 10.1186/1471-2288-12-3

**Published:** 2012-01-10

**Authors:** Alejandro Lazo-Langner, Marc A Rodger, Nicholas J Barrowman, Tim Ramsay, Philip S Wells, Douglas A Coyle

**Affiliations:** 1 Department of Medicine, University of Western Ontario, 800 Commissioners Rd E, London ON, N6A 5W9, Canada; 2 Department of Oncology, University of Western Ontario, 800 Commissioners Rd E, London ON, N6A 5W9, Canada; 3 Department of Epidemiology and Biostatistics, University of Western Ontario, 800 Commissioners Rd E, London ON, N6A 5W9, Canada; 4 Department of Medicine, University of Ottawa, 501 Smyth Road, Ottawa ON, K1H 8L6, Canada; 5 Department of Epidemiology and Community Medicine, University of Ottawa, 451 Smyth Road, Ottawa ON, K1H 8M5, Canada; 6 Clinical Epidemiology Program, Ottawa Health Research Institute, 725 Parkdale Ave, Ottawa ON, K1Y 4E9, Canada; 7 Chalmers' Research Group, Children's Hospital of Eastern Ontario Research Institute, 401 Smyth Rd, Ottawa ON, K1H 8L1, Canada

**Keywords:** Risk-Benefit Analysis, Decision Making, Meta-Analysis, Methods, Monte Carlo Method, Risk, indirect comparison

## Abstract

**Background:**

To demonstrate the use of risk-benefit analysis for comparing multiple competing interventions in the absence of randomized trials, we applied this approach to the evaluation of five anticoagulants to prevent thrombosis in patients undergoing orthopedic surgery.

**Methods:**

Using a cost-effectiveness approach from a clinical perspective (i.e. risk benefit analysis) we compared thromboprophylaxis with warfarin, low molecular weight heparin, unfractionated heparin, fondaparinux or ximelagatran in patients undergoing major orthopedic surgery, with sub-analyses according to surgery type. Proportions and variances of events defining risk (major bleeding) and benefit (thrombosis averted) were obtained through a meta-analysis and used to define beta distributions. Monte Carlo simulations were conducted and used to calculate incremental risks, benefits, and risk-benefit ratios. Finally, net clinical benefit was calculated for all replications across a range of risk-benefit acceptability thresholds, with a reference range obtained by estimating the case fatality rate - ratio of thrombosis to bleeding.

**Results:**

The analysis showed that compared to placebo ximelagatran was superior to other options but final results were influenced by type of surgery, since ximelagatran was superior in total knee replacement but not in total hip replacement.

**Conclusions:**

Using simulation and economic techniques we demonstrate a method that allows comparing multiple competing interventions in the absence of randomized trials with multiple arms by determining the option with the best risk-benefit profile. It can be helpful in clinical decision making since it incorporates risk, benefit, and personal risk acceptance.

## Background

In daily clinical practice clinicians are frequently presented with multiple competing treatment alternatives for the same clinical situation. In general it is accepted that when comparing several therapeutic alternatives the best evidence is derived from randomized trials. However, randomized trials are usually conducted comparing only two (or seldom three) options because inclusion of more treatment groups would require prohibitively large sample sizes and substantial increases in research costs and as a consequence, studies comparing all available treatment options at the same time are usually lacking.

Some notions should be noted regarding clinical decisions. First, if two or more equally effective alternatives are available for a disease the preferred one should be that conveying the lesser risk. Conversely, if all alternatives have equal risk, clinicians should opt for the most beneficial. Second, an alternative is usually not chosen if the risk associated with it outweighs the expected benefit [1]. The decision of using a specific option ultimately represents a personal choice and depends on the decision maker's perception of the associated benefits and risks with the potential caveat that such perception depends on factors such as the clinician's experience, knowledge, and expertise. Furthermore, when multiple alternatives exist for the same situation, the risks and benefits associated with each alternative should be pondered before deciding for a particular option.

Risks and benefits from medical interventions should be assessed together because their occurrence is inter-related and ideally, several factors should be considered in the decision process including: the expected clinical benefits and risks for each alternative, the uncertainty of these parameters, and the clinician's personal preference regarding the trade-off between benefits and risks [1,2]. A problem with this framework is that simultaneously incorporating all of the aforementioned factors for each available option in the decision process while at the same time considering all available evidence is practically difficult and not straightforward. Furthermore, since there are variations in personal preference for the trade-off between risks and benefits, comparative risk-benefit summaries should be presented throughout a range of trade-off values.

**Figure 1:**
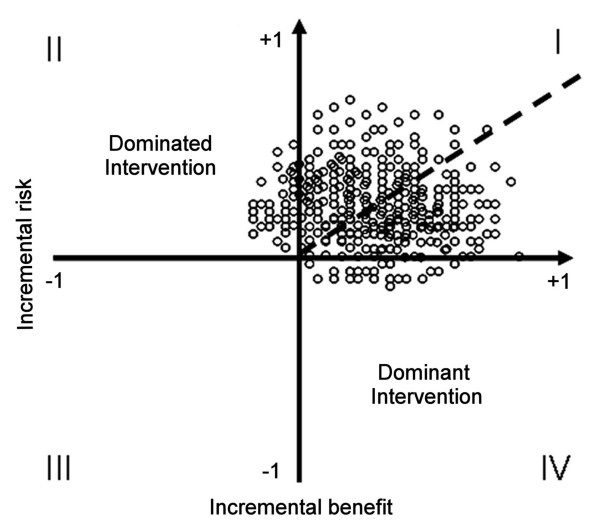
**Risk-benefit plane showing a hypothetical conjoint risk-benefit analysis**. In this hypothetical example, each point represents a joint risk-benefit observation calculated from a replication obtained from the Monte Carlo simulation. The percentage of the observations lying below the risk-benefit acceptability threshold (solid line) represents the probability of the intervention being net-beneficial for that specific threshold. It can be noted that a higher value of the threshold (i.e. a higher risk acceptance) will result in a higher probability of the intervention being net risk-beneficial.

There has been an increasing interest in methods for jointly assessing risk and benefit and some work has been done in this area [3-7]. Proposed approaches to evaluate benefit and risk include using the number needed to treat and the number needed to harm [4,5,8]. These estimations are generally used independently and they are seldom combined in a risk-benefit ratio. Major problems with this approach are: a) the risk-benefit ratio is difficult to interpret and extrapolate outside the trial generating the information, and b) assessing uncertainty of a ratio is difficult because estimation of confidence intervals is problematic [9-11]. Additionally, besides being conceptually difficult to interpret, these approaches have the disadvantage of not allowing a comparison between multiple agents when these are available for the same indication. Methods using meta-analytical techniques have been developed to indirectly compare interventions that have not been directly compared; however, these methods involve a comparison of effect sizes (e.g. odds ratios) and result in estimates with wide confidence intervals which are difficult to interpret and do not provide a conjoint assessment of benefits and risks [12,13]. A possible way to solve some of the aforementioned problems that takes advantage of the Bayesian framework is the use of modeling techniques that allow introducing a term of uncertainty around a parameter estimate using strategies such as Monte Carlo simulation or non-parametric bootstrapping [11,14-16]. In 2004 Lynd and O'Brien proposed an interesting approach to this problem [2]. Their method used a cost-effectiveness approach similar to the one used in the health economics literature [17] and involves the estimation of the joint density of incremental risk and incremental benefit using probabilistic simulation, which is then plotted in a risk-benefit plane analogous to the cost-benefit plane used in economics (Figure 1)[18]; then a risk-benefit acceptability curve (RBAC) is created. This curve shows the proportion of net risk-beneficial interventions at a given threshold for risk acceptance and is analogous to the cost-effectiveness acceptability curve [19]. A limitation of this method is that each curve compares only two interventions and, although multiple curves can be superimposed in a single plot, this will only inform the probability for each intervention of being risk-beneficial but it would not allow determining which one has the best risk-benefit profile.

Herein we elaborate on an extension of the method proposed by Lynd and O'Brien [2] in order to compare multiple competing interventions. We use information obtained from a meta-analysis of the literature to estimate the clinical risk (i.e. the "clinical cost") and benefit of each intervention together with an estimation of their corresponding uncertainties and, given that the optimal cutoff value for the tradeoff between risks and benefits is usually not known, we present the information through a range of tradeoff values which allows incorporating personal preference for risk acceptance. Finally, we incorporate a reference tradeoff value based on the clinical relevance of risk and benefit.

## Methods

It is well known that major orthopedic surgery, including total knee or hip arthroplasty, is associated with an increased risk of venous thromboembolic disease (VTE) -comprising deep vein thrombosis and pulmonary embolism- that might be as high as 85% and that anticoagulants decrease this risk but also result in increased bleeding events [20]. Given the high case-fatality rate of major bleeding avoiding an excess bleeding risk is as important as achieving a good preventive effect. In addition, anticoagulants have very few side effects other than hemorrhage and thus their risk and benefit can be readily defined. Several anticoagulant drugs are available for this indication but they all differ in their efficacy and safety and therefore, in order to define the best intervention the ideal study should include 5 or 6 arms making it unfeasible. We used a risk-benefit analysis framework as outlined in the following sections to compare five different anticoagulants for the prevention of VTE in patients undergoing major orthopedic surgery.

### Estimation of risks and benefits

While measuring the clinical benefit derived from an intervention is usually straightforward, defining the risk can be more problematic. Risk should be defined by an intervention-derived adverse event. Ideally, the intervention should have only one or very few adverse effects, of which the most frequent should be used to define the clinical cost. Alternatively, a composite endpoint can be used to incorporate different adverse events into a single outcome measure (e.g. mortality due to all causes or permanent disability). Since the risk and the benefit are defined by clinical events, it is necessary to know the proportion of patients experiencing such events. This can be obtained from a systematic review and meta-analysis of published evidence, which if properly conducted should provide a relatively unbiased summary of the available information [21].

To demonstrate the use of the methods proposed herein we use data from a previously conducted meta-analysis evaluating the use of anticoagulants for prophylaxis of VTE in patients undergoing major orthopedic surgery [22]. We included randomized controlled trials evaluating short-term (< 15 days) administration of anticoagulants for VTE prophylaxis in patients undergoing total hip or knee arthroplasty. The main benefit outcome was the proportion of major VTE (proximal deep vein thrombosis, pulmonary embolism or death) assessed by previously validated criteria [23]. The risk was assessed by estimating the proportion of major bleeding using a standard definition [24]. The search included MEDLINE, EMBASE, The Cochrane Library and also grey literature and was included studies published between 1980 and 2005. Details on the search strategy are available from the authors upon request.

The retrieved references were evaluated for inclusion independently by 2 reviewers and discrepancies were resolved by consensus. Data was abstracted by one reviewer and independently verified by a second reviewer. Quality of the studies was assessed using the criteria proposed by Jadad [25] and allocation concealment was evaluated according to the definition proposed by Schulz and Grimes [26]. A meta-analysis of proportions was conducted to obtain pooled estimates of proportions and their variances using a fixed or random effects model as described in Appendix 1. To determine the appropriate statistical model to be used, heterogeneity of the proportions across individual studies was calculated using a χ^2 ^statistic for a *k *× 2 table, considering as statistically significant a *p *< 0.1. Individual subgroup and sensitivity analyses were planned *a priori *for type of surgery, adequacy of allocation concealment, source of funding, quality score, blinded outcome adjudication, timing of initiation of anticoagulation and type of analysis. Publication bias was explored plotting point estimates versus precision, or alternatively sample size. All the analyses were done using Excel XP version (Microsoft Corp., Redmond WA) with the statistical add-in software package Analyse-it release 1.7 (Analyse-it Software, Leeds UK).

### Estimation of uncertainty, joint risk-benefit and risk-benefit analysis

To determine the risk-benefit profile of a therapeutic option it is necessary to determine the difference in the risk (i.e. the incremental risk or *ΔR*), the difference in the benefit (i.e. the incremental benefit or *ΔB*), and the ratio of both (i.e. the incremental risk-benefit ratio or IRBR) that such option has compared to a reference, usually placebo or the standard of treatment. The IRBR is analogous to the incremental cost-effectiveness ratio (ICER) and it can be defined as *ΔR*/*ΔB *(i.e. the ratio of the difference in risk to the difference in benefit between two competing treatments) representing a joint risk-benefit measure which can be plotted in a risk-benefit plane (Figure 1). An intervention will be dominant if it provides more benefit with less risk, and it will be dominated if it provides less benefit with more risk. Problem arises when interventions provide an increase in benefit with an associated increase in risk. In such cases the choice will depend on the decision maker's willingness-to-accept the risk. This is called the risk-benefit acceptability threshold (RBAT) and is represented by the slope of a line crossing the origin of the risk-benefit plane (Figure 1).

The use of the IRBR poses the problem of evaluating the uncertainty around the joint distribution. Uncertainty should be presented to avoid the ambiguity supposed by the use of the ratio alone [11]. Under the Bayesian framework proposed by Lynd and O'Brien, *ΔR *and *ΔB *are treated as random variables whose values lie over a specific distribution function, [2]. Since probabilities are limited by 0 and 1, their distribution is better expressed using the beta distribution which is defined on the interval (0, 1) and is frequently used as a prior distribution in Bayesian analysis. It is a continuous probability distribution with the probability density function defined by parameters *α *and *β*, where *α *is the expected number of subjects that will experience an event and *β *is the expected number of subjects that will not experience an event. In the case of Lynd and O'Brien, they used data from a single study to conduct further analysis, thus limiting the number of interventions evaluated to those included in the study used to obtain the information. However, the model can also be parameterized using the information derived from a systematic review and meta-analysis of the literature to estimate the probability of a patient experiencing an event, with the advantage of providing a more pragmatic estimate if the meta-analysis is well conducted.

We conducted risk-benefit analyses for each competing anticoagulant. Analyses were conducted from a clinical perspective and the analytic horizon was short since studies explored the occurrence of the clinical outcomes (VTE and bleeding) within 30 days of surgery, and therefore no discounting was considered. Using the method of moments [27] we calculated the α and β parameters of a beta distribution using the information obtained from the meta-analysis. Then the α and β values were used to parameterize a second order Monte Carlo simulation with 1,000 replications, each one representing a repeat estimation of the pooled proportion from the distribution previously defined. The simulations were done using Microsoft Excel 2002 (Microsoft Corp., Seattle WA) and CrystalBall 7·1 (Decisioneering Inc. Denver CO) software. Using the results of the Monte Carlo simulation, we calculated the percentage of the replications lying below a range of values for the RBAT. These values can be plotted in a risk-benefit acceptability curve, representing the probability of the intervention being risk-beneficial for a range of RBAT values. Although curves can be superimposed, they will only show the probability of each intervention being risk beneficial but will not inform which has the best risk-benefit profile.

### Calculation of the net clinical benefit and indirect comparison of multiple treatment options

Unfortunately, the mathematical properties of the IRBR yield some limitations: when the difference in effectiveness (or benefit) is small the confidence bounds may become too wide which makes it difficult to estimate the uncertainty of the ratio [28]. In economic analyses the use of the net monetary benefit has been proposed as an alternative to overcome the problems with the ICER [28]. Using a similar approach we calculated the net clinical benefit (NCB) which is analogous to the net monetary benefit and is a way to assign a clinical value to the increment in clinical benefit that is obtained from the new treatment, subtracting from this the increment in the risk derived from such treatment. If the NCB is positive it means that the risk of achieving an additional benefit is less than the value of the benefit achieved. If the NCB is negative then the treatment should not be accepted since the risk exceeds the value of the benefit achieved [29].

Using the probabilistic framework proposed by Lynd and O'Brien [2], we assumed that a treatment would be risk-beneficial if the IRBR is less than the RBAT represented by the linear expression

(1) ΔR∕ΔB<ρ

where Δ*R *denotes the differential risk, Δ*B *the differential benefit, and *ρ *is the RBAT. The inequality can be re-arranged as

(2) ρΔB-ΔR>0

or alternatively

(3) NCB=ρΔB-ΔR

with variance equal to

(4) $$\text{var}\left(NCB\right)=\rho^{2}\times \text{var}\left(\Delta B\right)+\text{var}\left(\Delta R\right)-2\times \text{cov}\left(\Delta B,\Delta R\right)$$

where *NCB *represents the net clinical benefit and for *NCB *to be positive *ρ*Δ*B *has to be greater than Δ*R*.

Taking advantage of this approach we extended Lynd and O'Brien's [2] method in order to compare multiple agents by evaluating which anticoagulant(s) result(s) in a greater clinical benefit when the risk associated with the intervention has been accounted for.

Using this approach we used data from the Monte Carlo simulations to calculate the *NCB_j _*for each *j *individual simulation trial using the equation

(5) $$NCB_{j}=\rho \Delta B_{j}-\Delta R_{j}$$

where Δ*R_j _*is the incremental risk and Δ*B_j _*is the incremental benefit obtained in the *j *individual simulation trial. The variance can be used to estimate 95 percent confidence limits if we assume a normal distribution of the data, or alternatively in order to avoid an assumption of normality we can estimate these limits using the values for the 2.5 and 97.5 percentiles of the values obtained from the simulation.

The *NCB_j _*was calculated for each simulation trial across a range of *ρ *values for each of the five anticoagulant agents included in the systematic review and for placebo. For each trial and each value of *ρ *we determined which anticoagulant had the highest *NCB_j_*. We then calculated for each agent and value of *ρ *the proportion of trials in which such agent had the highest *NCB_j_*. This proportion represents the probability that each anticoagulant had of achieving the highest net clinical benefit across a range of *ρ*values. The results were then plotted in a net clinical benefit probability curve which shows which of the competing treatment alternatives is/are most likely to have the best risk-benefit profile at a given RBAT value.

### Determination of a reference RBAT

Since the ideal RBAT is usually not known this analytic approach allows incorporating clinicians' personal risk acceptance in the decision process. However, the optimal RBAT depends on the relative weigh of the risk versus the benefit. However, determining the weighs can be problematic particularly if the outcomes of risk and benefit are surrogate markers. In the latter case, determining an outcome common to both risk- and benefit-defining events is necessary. In this work we obtained pooled estimates of the case fatality rates for both VTE and major bleeding and their 95% confidence intervals using the approach shown in appendix I. Finally, we calculated the case fatality rate ratio of VTE versus bleeding. These ratio indicates the relative importance of VTE to bleeding, in other words it informs how lethal is a thrombotic event compared to a bleeding event. To obtain the 95% CI for each ratio, a Monte Carlo simulation was conducted using 1,000 replications of the case fatality rates and ratios were calculated for each replication and the 2.5^th ^and 97.5^th ^percentile values of the replications were used to define the 95% confidence interval.

## Results

### Systematic review, estimation of risks, benefits, and case fatality rates

The search of the literature identified 1,583 potentially relevant citations of which 203 were fully assessed and 55 were included in the final review. These references are included in additional file 1. The reasons for exclusion and a flow diagram of the review are shown in the supplementary figure S1 included in additional file 1. The characteristics of the 55 included studies are shown in supplementary tables S1 and S2 included in additional file 1. We included studies evaluating, low molecular weight heparin (LMWH), unfractionated heparin (UFH), warfarin, fondaparinux, and ximelagatran, and placebo. The included studies comprised 123 intervention arms 2 of which were excluded. One study included a danaparoid arm in addition to the tinzaparin and dalteparin arms; the other study included an indomethacin arm together with a nadroparin and a placebo arms. In total 121 patient groups enrolling 42,131 patients were included in the review. Of these, 24,630 underwent total hip replacement, 13,318 underwent total knee replacement, 2,001 underwent surgery for hip fracture, and in 2,182 the type of surgery was not specified. Of the total number of patients enrolled in all the studies, 34,209 (81.2%) were evaluable for major VTE and 40,975 (97.3%) for major bleeding. The methodological characteristics of the studies are shown in the supplementary table S2 included in additional file 1. In general the methodological quality of the reports was acceptable. Allocation concealment was appropriate in approximately two thirds of the studies. The majority of the studies were funded by the pharmaceutical industry, and most of them used a blinded process to adjudicate outcomes. Only about one half of the studies used a similar definition for major bleeding events.

The pooled estimates of VTE and bleeding for all studies are shown in table 1. The pooled estimates were obtained using a random effects model. The drugs resulting in the highest and lowest proportion of major VTE were UFH and fondaparinux, respectively whereas those resulting in the highest and lowest proportion of bleeding were fondaparinux and warfarin, respectively. Sensitivity analyses showed that only the type of surgery influenced the occurrence of outcomes and therefore 2 separate analyses for patients undergoing total hip and total knee replacement were conducted. The random effects pooled estimates of the proportion of fatal events (case fatality rates) of major VTE and major bleeding were 1.391% (95% CI 0.892, 2.162) and 3.557% (95% CI 3.203, 3.911), respectively. Finally, the case fatality rate-ratio of major VTE with respect to major bleeding was 0.391 (95% CI 0.158, 1.579). In other words, in these studies on average, a major VTE was less lethal than a major bleeding event.

**Table 1:** Pooled estimates of proportions for risk and benefit outcomes in studies evaluating the use of anticoagulant prophylaxis for venous thromboembolism in orthopedic surgery

*Drug*	*Major Venous Thromboembolism*		*Major Bleeding*	
	***% (95% CI)***	***Variance***	***% (95% CI)***	***Variance***

**All patients**				

**Ximelagatran**	3.274 (3.175, 3.372)	0.098	1.804 (1.722, 1.885)	0.082

**LMWH**	6.528 (6.357, 6.699)	0.171	2.208 (2.156, 2.260)	0.052

**UFH**	13.394 (12.862, 13.926)	0.532	2.494 (2.363, 2.625)	0.131

**Warfarin**	6.278 (6.092, 6.463)	0.186	1.778 (1.690, 1.867)	0.088

**Fondaparinux**	2.051 (1.957, 2.146)	0.094	5.113 (4.690, 5.536)	0.423

**Placebo**	21.019 (19.978, 22.060)	1.041	1.781 (1.651, 1.912)	0.130

**Total hip replacement**				

**Ximelagatran**	3.401 (3.181, 3.621)	0.220	2.892 (2.683, 3.101)	0.209

**LMWH**	6.472 (6.293, 6.651)	0.179	2.151 (2.085, 2.216)	0.066

**UFH**	15.154 (14.446, 15.862)	0.708	2.813 (2.625, 3.001)	0.188

**Warfarin**	4.280 (4.083, 4.477)	0.197	2.229 (2.088, 2.371)	0.141

**Fondaparinux**	2.138 (2.009, 2.267)	0.129	6.033 (5.501, 6.566)	0.532

**Placebo**	24.726 (23.268, 26.184)	1.458	1.463 (1.299, 1.628)	0.164

**Total knee replacement**				

**Ximelagatran**	3.100 (3.042, 3.158)	0.058	1.127 (1.095, 1.159)	0.032

**LMWH**	5.143 (5.010, 5.277)	0.134	1.605 (1.566, 1.644)	0.039

**UFH**	9.119 (8.803, 9.436)	0.316	0.943 (0.837, 1.050)	0.106

**Warfarin**	8.100 (7.879, 8.322)	0.221	0.822 (0.792, 0.852)	0.030

**Fondaparinux**	2.446 (2.288, 2.603)	0.158	2.128 (2.003, 2.252)	0.124

**Placebo**	14.833 (14.351, 15.314)	0.482	2.116 (1.911, 2.322)	0.205

VTE Venous thromboembolism; CI Confidence interval; LMWH Low molecular weight heparin; UFH Unfractionated heparin.

### Estimation of uncertainty and risk-benefit analysis

**Figure 2:**
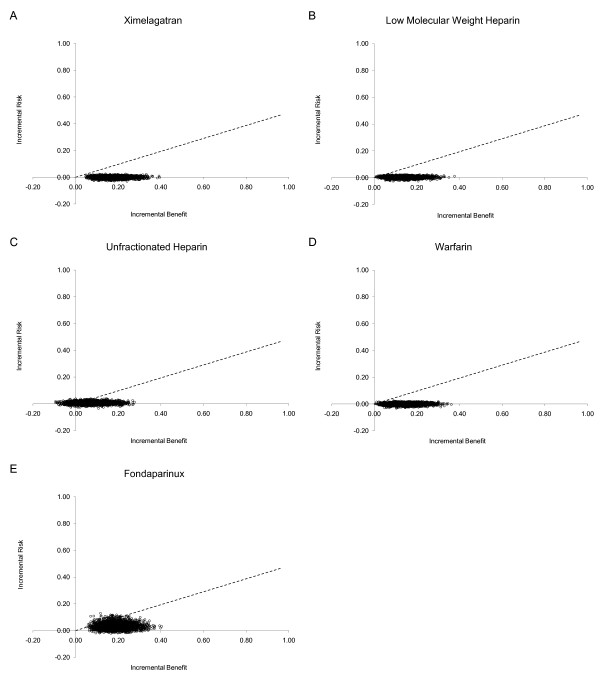
**Risk-benefit planes showing the joint incremental risk and benefit of anticoagulants used for venous thromboembolism prophylaxis in orthopedic surgery**. The figure shows separate analyses for patients receiving ximelagatran (A), low molecular weight heparin (B), Unfractionated heparin (C), warfarin (D) and fondaparinux (E). The dashed line corresponds to the reference value for the risk benefit acceptability threshold.

**Figure 3:**
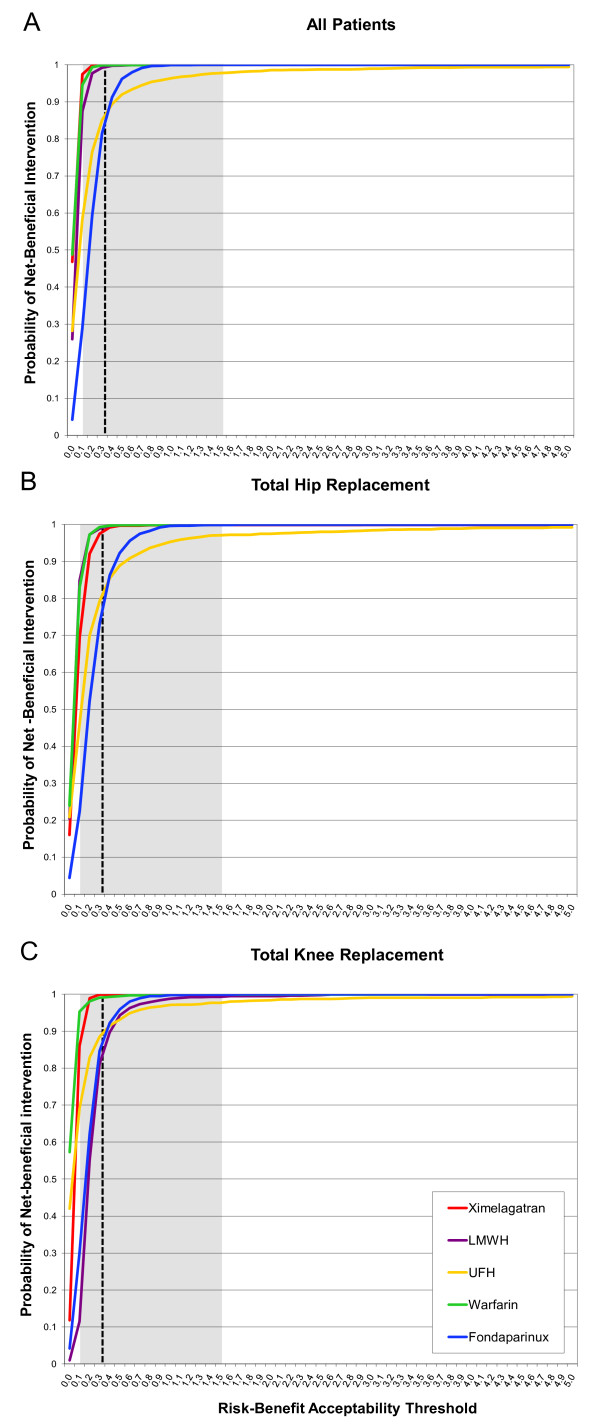
**Risk-benefit acceptability curves for anticoagulants used for venous thromboembolism prophylaxis in orthopedic surgery compared to placebo for all patients (A), total hip replacement patients (B) and total knee replacement patients (C)**. The curves show for each anticoagulant the probability of being net risk-beneficial compared to placebo across different risk-benefit acceptability thresholds which reflect the willingness to accept the risk of major bleeding episodes. The reference value is shown as the vertical dashed line with the 95% confidence interval shown as the shadowed area. Note that these curves do not allow a comparison of all agents simultaneously and do not inform the option with the best risk-benefit profile.

**Figure 4:**
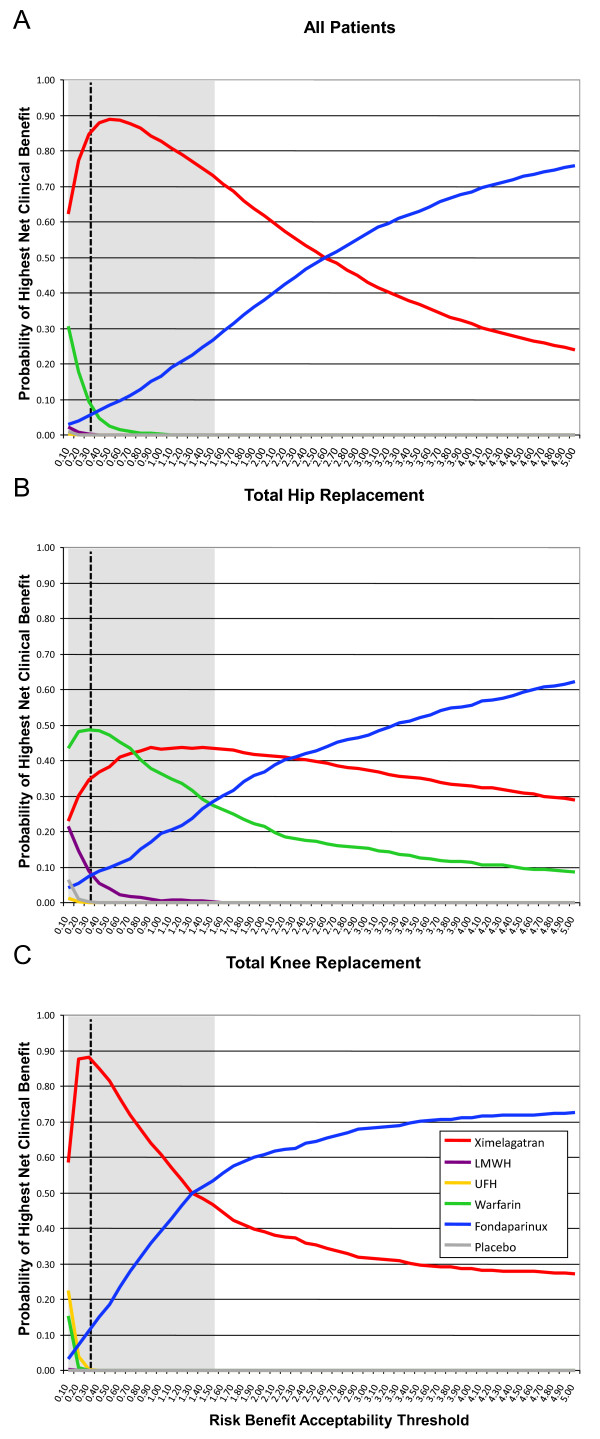
**Net clinical benefit probability curves for anticoagulants used in the prevention of venous thromboembolism in all patients undergoing orthopedic surgery (A), total hip replacement (B) and total knee replacement (C)**. The curves plot the probability that each drug has of providing the highest net clinical benefit -and consequently having the best risk-benefit profile- for each value of the risk-benefit acceptability threshold. The reference value is shown as the vertical dashed line with the 95% confidence interval shown as the shadowed area. Note that the probabilities change at different values of risk acceptance and that at each value the probabilities add up to one.

The information on risks and benefits obtained from the systematic review was used to parameterize Monte Carlo simulations. The results of the simulations were used to calculate the incremental benefits and risks for all anticoagulants which were subsequently used to construct risk-benefit planes. (Figures 2A-E). The proportion of paired observations lying below the risk-benefit acceptability threshold -represented by a dashed line in Figure 2- was calculated across a range of values and plotted in risk-benefit acceptability curves for each agent that were overlapped in a single plot (Figure 3A). Separate analyses were conducted for patients undergoing total hip or knee replacement (Figures 3B and 3C). These curves represent the probability that an agent has of being net-beneficial relative to placebo across a range of values for the risk-benefit acceptability threshold. In all analyses, as expected all options had high probabilities of being net-beneficial compared to placebo (Proportion > 0.8 with an RBAT = 0.391). The probabilities did not differ in subgroup analyses according to surgery type (total hip or knee replacement). However, as can be seen in Figure 3, the risk-benefit acceptability curves do not allow a comparison of all options at the same time. Thus, in order to compare all agents we calculated the NCB for each one of the 1000 simulations and subsequently we calculated for each agent the proportion of the simulated trials in which each agent had the highest NCB across a range of RBAT values. Results were plotted as probabilities in net clinical benefit probability curves (Figures 4A-C). These curves show which drug(s) has(have) the highest probability of having the highest net clinical benefit (and this the best risk-benefit profile) at a given value of the RBAT. As depicted in Figure 4A, ximelagatran was the agent with the highest probability (87.8%) of having the best net-clinical benefit when considering all patients, but subgroup analyses showed that this difference was mainly driven by the total knee replacement population (Figure 4C) in which the probability was 85.2% for ximelagatran, and that in the case of total hip replacement patients, the choice between warfarin or ximelagatran would be probably indifferent with probabilities of obtaining the highest net clinical benefit of 48.6% and 36.7%, respectively. It can be seen that the agents vary in their probability of having the best risk-benefit profile as the value of risk acceptance (RBAT) changes. Finally, because the optimal RBAT is not known, a reference value was calculated based on 'hard' outcomes. The clinical relevance of VTE relative to that of major bleeding was estimated using the values of the case fatality rate-ratios. These ratios and their 95% CI were then plotted in the curves to provide an estimate of the approximate level of risk acceptance (RBAT) that should be considered. The ratios were indicated in the curves as the vertical dashed line with the 95% CI indicated by the shaded area.

## Discussion

The present study demonstrates the use of risk-benefit analysis to perform indirect comparisons of multiple competing interventions. We believe that this approach might help to guide clinical decisions in areas where tension between risk and benefit exist and there are multiple therapeutic options. This approach provides a way to indirectly compare several interventions in the absence of randomized trials involving multiple arms.

This method assumes that the use of certain therapy has a clinical risk, something particularly important when potentially harmful therapies are used. Clinicians opt for these options because the expected benefit outweighs the risk; however, choosing the best therapeutic option can be difficult when the rates of major complications (risk) or clinical effectiveness (benefits) from different options vary. Furthermore, clinicians' willingness-to-accept the risks might differ depending on a number of issues. If for a clinical situation there are numerous therapeutic options it is unlikely that all options will be compared in a randomized trial usually because of sample size and research cost constraints. The approach described herein takes advantage of the risk-benefit analysis framework and provides a way to indirectly compare several interventions in order to determine the one with the best risk-benefit profile using information available from randomized trials to estimate costs and benefits.

Using the proposed approach we conducted an indirect comparison of five common anticoagulant drugs used to prevent the development of thrombotic complications in patients undergoing major orthopedic surgery. This intervention is particularly suited to exemplify this approach given that anticoagulants are associated with bleeding episodes as their major (and almost only) complication and with a palpable benefit, namely prevention of thrombosis. Furthermore, they are usually given for a short period of time which facilitates defining the time horizon for the study. The analysis showed that compared to placebo, all agents are likely risk-beneficial (Figures 3A-C), a finding that is not surprising. The problem is then choosing the one(s) with the best risk-benefit profile. By calculating the net clinical benefit for each anticoagulant at a particular value of the RBAT it can be easily determined the probability of obtaining the highest net clinical benefit for each competing anticoagulant at that particular RBAT value. These probabilities are then calculated for a range of RBAT values and used to create a net clinical benefit probability curve (Figures 4A-C). Furthermore, since the ultimate clinical consequences of major bleeding and venous thromboembolic events might be different (i.e. their associated mortality), we incorporated in the analysis a reference range of RBAT values derived from the case fatality rate-ratios of thrombosis and bleeding. The final plots then incorporate the key elements of a decision, namely risk, benefit, willingness to accept the risk and their uncertainties.

It can be argued that when analyzing and pooling information obtained from randomized trials, the use of proportions for comparison purposes -as opposed to effect sizes- supposes the loss of the randomization effect because groups are treated independently. Nevertheless, if properly conducted and reported, information derived from randomized trials has usually a high quality [30], and although we recognize that there might be a concern regarding the generalizability of the results from randomized trials, pooled estimates of event proportions will most likely approach reality as the population in the included studies increases. Although non-randomized studies could potentially also be used we prefer randomized trials which are less prone to bias. However, since a number of issues could influence meta-analysis results, systematic reviews should be methodologically sound and incorporate a priori all pertinent subgroup and sensitivity analyses, an evaluation of the homogeneity of outcomes' definitions used across different studies, and an assessment of study quality using validated scales [25,31]. This issue can be better appreciated in our study by analyzing the results for all patients and the subgroup analyses which showed that the risk-benefit profiles of the different anticoagulants were different in patients undergoing hip or knee replacement

Some conditions are necessary to conduct study using the approach proposed herein: First, outcomes used to measure risks and benefits must be defined similarly across studies. Incorporating studies using different outcome definitions would result in a heterogeneous result difficult to interpret. It is entirely possible that the results of the study example could be different if all and each one of the potential side effects of the drugs were incorporated in the analysis. Second, if clinically acceptable, risks and benefits should ideally be defined by a single outcome; if this is not possible then a composite outcome could be used, such as mortality. Third, events defining risks and benefits should be clinically relevant. Fourth, if the systematic review spans several years, it is particularly important to test for secular trends since outcome definitions might change over time as a result of new knowledge, which might affect the evaluation of older therapies still in use. In addition, changes in medical or surgical techniques or in health policy are likely to influence the results and therefore populations might not be similar. It can be appreciated that the systematic review must be stringently rigorous and adhere to standardized requirements such as the PRISMA (formerly QUOROM) statement and exploring heterogeneity becomes essential. A thorough sensitivity analysis should be conducted prior to incorporating the information into the Monte Carlo simulation, to assure to the maximum extent that studies include comparable populations. If these conditions are met, it can be argued that this approach provides a pragmatic panorama of the situation studied.

Our approach does not estimate effect sizes; instead it provides the clinician with information regarding the probability of having the best risk-benefit profile that certain intervention will have at a given value of risk acceptance. If risks and benefits can be measured in equivalent terms (e.g. deaths induced or prevented by the treatment), the preferred choice should be that conferring the highest probability of being beneficial at the chosen value for risk acceptance. However, if surrogate endpoints are used case fatality rates might be different and so will be the clinical relevance of costs and benefits relative to one another. Our method allows for an adjustment by changing the value of the RBAT to one that better suits the clinical situation being studied. Since the level of risk acceptance is pivotal to making the best choice, a reference value for the RBAT can be obtained from expert consensus or surveys, or by using case-fatality rate ratios. In any case a reference RBAT should be a guideline for the clinician to be applied on an individual basis.

It is important to note that randomized trials are usually powered to detect differences in benefit-defining events and inadequately powered to detect differences in risk and only some of them include risk-defining events in a composite outcome. In this regard, an advantage of our approach is that it allows larger sample sizes; hence more power to detect differences in less common, more clinically meaningful outcomes.

The potential limitations of our approach arise from three issues: 1) the fact that the information is obtained retrospectively from a systematic review and meta-analysis with their inherent caveats; 2) the methodological issues regarding the pooling of single proportions; and 3) the limitations regarding modeling techniques and their application to clinical risk-benefit ratio and incremental risk-benefit ratio analysis. With respect to the first point we considered that an ample sensitivity analysis should establish the robustness of the conclusions. If the conclusions are not robust, the conduction of a study using such information should probably be questioned.

In regard to the second point there are examples of the application of the proposed pooling techniques [32] and a major strength of this method is the fact that the estimates are the result of a comprehensive review and meta-analysis including all major studies. A potential problem with the weighting method of Laird and Mosteller is that it uses a normal approximation to proportions which might be problematic if the proportions are very small because they might not have a normal distribution anymore [33]. In a similar fashion, the statistical properties of a χ2 test to determine heterogeneity might be adversely affected when proportions are very small. If this is the case, robustness might be assessed by using alternate pooling methods or comparing fixed versus random approaches. Finally, although the modeling techniques used in this study are not commonly found in clinical medicine and their application to clinical decision making was described only very recently hey are frequent in the economic literature and have been well validated. Additionally, other concerns have been recently raised regarding the use of cost-effectiveness analysis (in our case risk-benefit analysis), namely the degree of discrepancy between probability-based and expectation-based methods, as well as non-transitivity in pair-wise comparisons [34,35]. These problems could potentially arise in economic studies and their potential effects on studies using the approach proposed herein deserves further evaluation. Finally, the performance of the present approach compared to other methods for indirect comparisons still remains to be tested.

## Conclusions

Herein we demonstrate the application of risk-benefit analysis to conduct indirect comparisons of competing interventions using a Bayesian framework and incorporating the key elements of the decision process (rIsk, benefit, and willingness-to-accept the risk) and might prove to be a valuable aid in order to facilitate the implementation and practice of evidence-based medicine.

## List of abbreviations

**ICER: **Incremental cost-effectiveness ratio; **IRBR: **Incremental risk-benefit ratio; **LMWH: **Low molecular weight heparin; **NCB: **Net clinical benefit; **RBAC: **Risk-benefit acceptability curve; **RBAT: **Risk-benefit acceptability threshold; **UFH: **Unfractionated heparin.

## Competing interests

The authors declare that they have no competing interests.

## Authors' contributions

AL-L participated in the conception, design, and development of the method, as well as in data collection, analysis, and drafting of the manuscript, MAR participated in the development of the method, as well as in data collection, analysis, and drafting of the manuscript, NJB participated in the development of the statistical methods and drafting of the manuscript, TR participated in the development of the statistical methods and drafting of the manuscript, PSW participated in data collection, analysis, and drafting of the manuscript, and DAC participated in the conception, design, and development of the method, as well as in analysis, and drafting of the manuscript. All authors read and approved the final manuscript.

## Appendix 1

### Methodology for the calculation of pooled estimates of proportions

For a collection of *k *studies, each *i *individual study giving a *p_i _*proportion

(6) $$p_{i}=x_{i}/)n_{i}$$

where *x_i _*is the number of events and *n_i _*the number of patients in the *i*^th ^study; for *i = *1 to *k*

(7) $$n=\sum_{i=1}^{k}n_{i}$$

and

(8) $$x=\sum_{i=1}^{k}x_{i}$$

If homogeneity holds, a $\hat{p}$estimate of the true probability is

(9) $$\hat{p}=\frac{\sum_{i=1}^{k}x_{i}}{\sum_{i=1}^{k}n_{i}}$$

with variance

(10) $$\text{var}\left(\hat{p}\right)=\frac{\hat{p}\left(1-\hat{p}\right)}{\sum_{i=1}^{k}n_{i}}$$

The confidence interval can be calculated using the Wilson score method [36] as follows:

(11) $$\frac{\left(2np+z^{2}\pm z\sqrt{\left(z^{2}+4npq\right)}\right)}{2\left(n+z^{2}\right)}$$

where *z *is the 1 - α/2 point of the standard Normal distribution, and *q *= 1 - *p*.

If the proportions are not homogeneous then a random effects estimator ${\hat{\theta }}_{R}$ of the true proportion may be defined as

(12) $${\hat{\theta }}_{R}=\frac{\sum_{i=1}^{k}w_{i}^{*}p_{i}}{\sum_{i=1}^{k}w_{i}^{*}}$$

with variance

(13) $$\text{var}\left({\hat{\theta }}_{R}\right)={\left(\sum_{i=1}^{k}w_{i}^{*}\right)}^{-1}$$

where the weights $w_{i}^{*}$are defined as proposed by Laird and Mosteller [33]

(14) $$w_{i}^{*}=\frac{1}{\left(\bar{p}\left(1-\bar{p}\right)-{\hat{\tau }}^{2}\right)∕n_{i}+{\hat{\tau }}^{2}}$$

where the mean proportion $\bar{p}$for the *k *collection of individual *i *studies is

(15) $$\bar{p}=\sum_{i=1}^{K}p_{i}∕k$$

and where the ${\hat{\tau }}^{2}$ estimate of the variance of the proportions is

(16) $${\hat{\tau }}^{2}=\frac{{\sum_{i=1}^{K}\left(p_{i}-\bar{p}\right)}^{2}}{k-1}-\frac{\sum_{i=1}^{K}p_{i}\left(1-p_{i}\right)∕n_{i}}{k}$$

## Pre-publication history

The pre-publication history for this paper can be accessed here:

http://www.biomedcentral.com/1471-2288/12/3/prepub

## Supplementary Material

Additional file 1 **Supplementary information of the systematic review**. This file contains the following information regarding the systematic review: flow diagram showing the progress of the systematic review (figure), characteristics of the studies included in the systematic review (table), methodological characteristics of the studies included in the systematic review (table), and references of the studies included in the systematic review. Click here for file
