# Supplementary material for: Comparing multiple competing interventions in the absence of randomized trials using clinical risk-benefit analysis
Source: BMC Med Res Methodol. 2012 Jan 10;12:3. doi: 10.1186/1471-2288-12-3 (PMC3292458; doi:10.1186/1471-2288-12-3)
Supplement: Additional file 1 — Supplementary information of the systematic review. This file contains the following information regarding the systematic review: flow diagram showing the progress of the systematic review (figure), characteristics of the studies included in the systematic review (table), methodological characteristics of the studies included in the systematic review (table), and references of the studies included in the systematic review. [file 1471-2288-12-3-S1.DOC]

**Comparing multiple competing interventions in the absence of randomized trials using clinical risk-benefit analysis.** Alejandro Lazo-Langner, Marc A. Rodger, Nicholas J. Barrowman, Tim Ramsay, Philip S. Wells, Douglas A. Coyle

**Supplementary materials**

***Supplementary Figure 1.*** *Flow diagram showing the progress of the systematic review*

| Supplementary Table 1. Characteristics of the studies included in the systematic review | | | | | | | | |
| --- | --- | --- | --- | --- | --- | --- | --- | --- |
| *Year / Author / Reference* | *Jadad Score* | *Allocation Concealment* | *Funding* | *Outcome Allocation* | *Follow up (days)* | *Type of Procedure* | *No. of Randomized Patients* | *Interventions* |
| 1983 Leyvraz^a^ [1] | 3 | Adequate | Pharmaceutical | Blinded | ≤15 | THR | 96 | UFH 3,500 IU TID Pre. |
| 1986 Turpie[2] | 5 | Inadequate / Unclear | Non Pharmaceutical / Unclear | Blinded | ≤15 | THR | 100 | Enoxaparin 30 mg BID Post.  Placebo |
| 1988 Planes[3] | 4 | Inadequate / Unclear | Non Pharmaceutical / Unclear | Blinded | ≤15 | THR | 237 | Enoxaparin 40 mg OD Pre.  UFH 5,000 IU TID Peri. |
| 1989 Monreal[4] | 4 | Adequate | Non Pharmaceutical / Unclear | Not Blinded / Unclear | ≤15 | HFS | 90 | Dalteparin 5,000 IU anti-Xa OD Peri.  UFH 5,000 IU TID Peri. |
| 1991 Eriksson[5] | 4 | Adequate | Non Pharmaceutical / Unclear | Blinded | ≤15 | THR | 136 | Dalteparin 5,000 IU anti-Xa OD Pre.  UFH 5,000 IU TID Peri. |
| 1991 Lassen[6] | 4 | Inadequate / Unclear | Non Pharmaceutical / Unclear | Blinded | ≤15 | THR | 210 | Tinzaparin 50 IU anti-Xa /Kg OD Pre.  Placebo |
| 1991 Levine[7] | 5 | Inadequate / Unclear | Non Pharmaceutical / Unclear | Blinded | ≤15 | THR | 665 | Enoxaparin 30 mg BID Post.  UFH 7,500 IU BID Post. |
| 1991 Leyvraz^b c^ [8] | 3 | Adequate | Pharmaceutical | Blinded | ≤15 | THR | 409 | Nadroparin 62 IU anti-Xa/Kg Pre.  UFH Approx. 4,000 IU TID Pre. |
| 1991 Torholm[9] | 4 | Inadequate / Unclear | Non Pharmaceutical / Unclear | Not Blinded / Unclear | 42 | THR | 112 | Dalteparin 5,000 IU anti-Xa OD Peri.  Placebo |
| 1992 GHAT[10] | 5 | Adequate | Pharmaceutical | Blinded | 42 | THR | 335 | Nadroparin 10,000 IU anti-Xa OD Pre.  UFH 5,000 IU TID Pre. |
| 1992 Leclerc[11] | 5 | Adequate | Pharmaceutical | Blinded | 42 | TKR | 131 | Enoxaparin 30 mg BID Post.  Placebo |
| 1993 Hull[12] | 5 | Adequate | Pharmaceutical | Blinded | 90 | THR TKR | 1436 | Tinzaparin 75 IU anti-Xa/Kg OD Post.  Warfarin Target INR 2-3 Peri. |
| 1993 Platz[13] | 2 | Inadequate / Unclear | Non Pharmaceutical / Unclear | Not Blinded / Unclear | ≤15 | HFS | 68 | Certoparin 3,000 IU anti-Xa Pre.  UFH 5,000 IU TID Pre. |
| 1994 Colwell[14] | 2 | Adequate | Pharmaceutical | Not Blinded / Unclear | ≤15 | THR | 610 | Enoxaparin 40 mg OD Post.  Enoxaparin 30 mg BID Post.  UFH 5,000 IU TID Post. |
| 1994 Fauno[15] | 2 | Adequate | Pharmaceutical | Blinded | ≤15 | TKR | 224 | Enoxaparin 40 mg OD Pre.  UFH 5,000 IU TID Pre. |
| 1994 Friedman[16] | 3 | Inadequate / Unclear | Pharmaceutical | Blinded | ≤15 | THR TKR | 1173 | Ardeparin 50 IU anti-Xa/Kg BID Peri.  Ardeparin 90 IU anti-Xa/Kg OD Peri.  Warfarin Target INR 1.5-3.0 Pre. |
| 1994 Spiro[17] | 4 | Adequate | Pharmaceutical | Blinded | ≤15 | THR | 572 | Enoxaparin 10 mg OD Post.  Enoxaparin 40 mg OD Post.  Enoxaparin 30 mg BID Post. |
| 1995 Avikainen[18] | 1 | Inadequate / Unclear | Non Pharmaceutical / Unclear | Not Blinded / Unclear | ≤15 | THR | 167 | Enoxaparin 40 mg OD Pre.  UFH 5,000 IU BID Peri. |
| 1995 Colwell[19] | 2 | Inadequate / Unclear | Pharmaceutical | Blinded | ≤15 | TKR | 453 | Enoxaparin 30 mg BID Peri.  UFH 5,000 IU TID Peri. |
| 1995 Hamulyak[20] | 3 | Adequate | Pharmaceutical | Blinded | ≤15 | THR TKR | 672 | Nadroparin 60 IU anti-Xa/Kg OD Pre.  Acenocoumarol Target INR 2-3 Pre. |
| Supplementary Table 1. Characteristics of the studies included in the systematic review | | | | | | | | |
| *Year / Author / Reference* | *Jadad Score* | *Allocation Concealment* | *Funding* | *Outcome Allocation* | *Follow up (days)* | *Type of Procedure* | *No. of Randomized Patients* | *Interventions* |
| 1995 Lee[21] | 1 | Inadequate / Unclear | Non Pharmaceutical / Unclear | Not Blinded / Unclear | ≤15 | THR | 72 | Dalteparin 5,000 IU anti-Xa OD Peri.  Warfarin Target INR 2.5 Pre. |
| 1995 Warwick[22] | 2 | Inadequate / Unclear | Non Pharmaceutical / Unclear | Blinded | ≤15 | THR | 156 | Enoxaparin 40 mg OD Pre.  Placebo |
| 1996 Kalodiki^d^ [23] | 5 | Inadequate / Unclear | Pharmaceutical | Blinded | ≤15 | THR | 93 | Enoxaparin 40 mg OD Pre.  Placebo |
| 1996 Leclerc[24] | 5 | Adequate | Pharmaceutical | Blinded | 180 | TKR | 670 | Enoxaparin 30 mg BID Post.  Warfarin Target INR 2-3 Peri. |
| 1996 Levine^e^ [25] | 5 | Adequate | Non Pharmaceutical / Unclear | Blinded | ≤15 | TKR | 246 | Ardeparin 50 IU anti-Xa/Kg BID Post.  Placebo |
| 1996 Schwartsmann[26] | 1 | Inadequate / Unclear | Non Pharmaceutical / Unclear | Not Blinded / Unclear | ≤15 | THR | 99 | Enoxaparin 40 mg OD Peri.  UFH 5,000 IU TID Peri. |
| 1997 Francis[27] | 2 | Inadequate / Unclear | Non Pharmaceutical / Unclear | Blinded | ≤15 | THR | 580 | Dalteparin 5,000 IU anti-Xa OD Peri.  Warfarin Target INR 2.5 Pre. |
| 1997 Heit[28] | 5 | Adequate | Pharmaceutical | Blinded | ≤15 | TKR | 833 | Ardeparin 25 IU anti-Xa/Kg BID Peri.  Ardeparin 35 IU anti-Xa/Kg BID Peri.  Ardeparin 50 IU anti-Xa/Kg BID Peri.  Warfarin Target INR 2-3 Pre. |
| 1997 Yoo^b^ [29] | 1 | Inadequate / Unclear | Pharmaceutical | Not Blinded / Unclear | ≤15 | THR | 100 | Nadroparin 62 IU anti-Xa Kg OD Pre.  Placebo |
| 1998 Planes[30] | 4 | Adequate | Non Pharmaceutical / Unclear | Blinded | ≤15 | THR | 498 | Reviparin 4,200 IU anti-Xa OD Pre.  Enoxaparin 40 mg OD Pre. |
| 1999 Adolf[31] | 4 | Inadequate / Unclear | Pharmaceutical | Blinded | ≤15 | THR | 341 | Certoparin 3,000 IU anti-Xa OD Pre.  Certoparin 5,000 IU anti-Xa OD Pre. |
| 1999 Colwell[32] | 2 | Inadequate / Unclear | Pharmaceutical | Blinded | 90 | THR | 3011 | Enoxaparin 30 mg BID Post.  Warfarin Target INR 2-3 Post. |
| 1999 Planes[33] | 5 | Adequate | Pharmaceutical | Blinded | ≤15 | THR | 499 | Enoxaparin 40 mg OD Pre.  Tinzaparin 4,500 IU anti-Xa OD Pre. |
| 1999 TIFDED^f^ [34] | 2 | Inadequate / Unclear | Pharmaceutical | Blinded | 56 | HFS | 132 | Enoxaparin 40 mg OD Peri.  Dalteparin 5,000 IU anti-Xa OD Peri. |
| 2000 Hull^g^ [35] | 5 | Adequate | Pharmaceutical | Blinded | ≤15 | THR | 1472 | Dalteparin 5,000 IU anti-Xa OD Peri.  Warfarin Target INR 2-3 Peri. |
| 2000 Kakkar[36] | 4 | Adequate | Pharmaceutical | Blinded | 56 | THR | 298 | Bemiparin 3,500 IU anti-Xa OD Peri.  UFH 5,000 IU BID Peri. |
| 2000 Lassen[37] | 4 | Adequate | Non Pharmaceutical / Unclear | Blinded | 70 | THR | 250 | Tinzaparin 75 IU anti-Xa/Kg OD Pre.  Tinzaparin 50 IU anti-Xa Kg OD Peri. |
| 2001 Bauer[38] | 5 | Adequate | Pharmaceutical | Blinded | 49 | TKR | 1049 | Enoxaparin 30 mg BID Post.  Fondaparinux 2.5 mg OD Peri. |
| 2001 Eriksson[39] | 5 | Adequate | Pharmaceutical | Blinded | ≤15 | HFS | 1711 | Enoxaparin 40 mg OD Post.  Fondaparinux 2.5 mg OD Peri. |
| 2001 Fitzgerald[40] | 3 | Adequate | Pharmaceutical | Blinded | ≤15 | TKR | 349 | Enoxaparin 30 mg BID Peri.  Warfarin Target INR 2-3 Peri. |
| 2001 Haas[41] | 4 | Adequate | Pharmaceutical | Blinded | ≤15 | THR TKR | 2021 | Reviparin 4,200 IU anti-Xa OD Pre.  UFH 7,500 IU BID Pre. |
| Supplementary Table 1. Characteristics of the studies included in the systematic review | | | | | | | | |
| *Year / Author / Reference* | *Jadad Score* | *Allocation Concealment* | *Funding* | *Outcome Allocation* | *Follow up (days)* | *Type of Procedure* | *No. of Randomized Patients* | *Interventions* |
| 2001 Heit[42] | 3 | Adequate | Pharmaceutical | Blinded | 28 | TKR | 255 | Ximelagatran 24 mg BID Post.  Enoxaparin 30 mg BID Post. |
| 2001 Turpie[43] | 5 | Adequate | Pharmaceutical | Blinded | 42 | THR | 933 | Enoxaparin 30 mg BID Post.  Fondaparinux 0.75, 1.5, 3, 6, and 8 mg OD Peri. |
| 2002 Eriksson^h^ [44] | 5 | Adequate | Pharmaceutical | Blinded | ≤15 | THR TKR | 760 | Ximelagatran 24 mg BID Peri.  Dalteparin 5,000 IU anti-Xa OD Pre. |
| 2002 Eriksson^h^ [45] | 2 | Inadequate / Unclear | Pharmaceutical | Blinded | 49 | THR TKR | 67 | Ximelagatran 24 mg BID Peri.  Dalteparin 5,000 IU anti-Xa OD Pre. |
| 2002 Francis[46] | 5 | Adequate | Pharmaceutical | Blinded | ≤15 | TKR | 680 | Ximelagatran 24 mg BID Post.  Warfarin Target INR 2.5 Peri. |
| 2002 Lassen[47] | 5 | Adequate | Pharmaceutical | Blinded | 49 | THR | 2309 | Enoxaparin 40 mg OD Pre.  Fondaparinux 2.5 mg OD Peri. |
| 2002 Turpie[48] | 5 | Adequate | Pharmaceutical | Blinded | ≤15 | THR | 2275 | Enoxaparin 30 mg BID Post.  Fondaparinux 2.5 mg OD Peri. |
| 2003 Colwell[49] | 5 | Adequate | Pharmaceutical | Blinded | ≤15 | THR | 1838 | Ximelagatran 24 mg BID Pre.  Enoxaparin 30 mg BID Post. |
| 2003 Eriksson^h^ [50] | 5 | Adequate | Pharmaceutical | Blinded | 42 | THR TKR | 2764 | Ximelagatran 24 mg BID Peri.  Enoxaparin 40 mg OD Pre. |
| 2003 Eriksson^h^ [51] | 5 | Adequate | Pharmaceutical | Blinded | ≤15 | THR TKR | 2788 | Ximelagatran 24 mg BID Peri.  Enoxaparin 40 mg OD Pre. |
| 2003 Francis[52] | 5 | Adequate | Pharmaceutical | Blinded | ≤15 | TKR | 2301 | Ximelagatran 24 mg BID Post.  Ximelagatran 36 mg BID Post.  Warfarin Target INR 2.5 Peri. |
| 2003 Navarro-Quilis[53] | 5 | Adequate | Pharmaceutical | Blinded | 42 | TKR | 381 | Bemiparin 3,500 IU anti-Xa OD Peri.  Enoxaparin 40 mg OD Pre. |
| 2004 Wang^i^ [54] | 0 | Inadequate / Unclear | Non Pharmaceutical / Unclear | Blinded | ≤15 | TKR | 101 | Nadroparin 1,900-3,800 IU anti-Xa OD Pre.  Placebo |
| 2005 Colwell[55] | 5 | Adequate | Pharmaceutical | Blinded | 42 | TKR | 2303 | Ximelagatran 36 mg BID Post.  Warfarin Target INR 2.5 Peri. |
| GHAT German hip arthroplasty trial group; TIFDED Thromboprophylaxis in Fracture Surgery: Danaparoid, Enoxaparin, Dalteparin Study Group; THR Total hip replacement; TKR Total knee replacement; HFS Hip fracture surgery; IU anti-Xa International units anti-activated factor X; OD Once daily; BID Twice daily; TID Three times daily; UFH Unfractionated heparin; Pre Preoperative timing of initiation; Peri Perioperative timing of initiation; Post Postoperative timing of initiation; INR International normalized ratio  ^a^ This trial included two arms using the same initial dose; one arm had dosing adjustments according to activated partial thromboplastin time.  ^b^ The initial dose of nadroparin was 41 IU anti-Xa/Kg OD during the first 3 days.  ^c^ The unfractionated heparin arm was adjusted according to activated thromboplastin time.  ^d^ This trial included two enoxaparin groups one of which used graduate compression stockings.  ^e^ In this trial both groups used graduate compression stockings.  ^f^ This trial included a danaparoid arm.  ^g^ This trial included two dalteparin arms starting at 2 hours pre- and 4 hours postoperatively.  ^h^ These trials used initial subcutaneous melagatran in the ximelagatran arm.  ^i^ This trial included an indomethacin arm. | | | | | | | | |

| Supplementary Table 2. Methodological characteristics of the studies included in the systematic review | | |
| --- | --- | --- |
| *Characteristic* | *Number* | *Percentage* |
| **Jadad score** |  |  |
| 0 | 1 | 1.8 |
| 1 | 4 | 7.3 |
| 2 | 9 | 16.4 |
| 3 | 6 | 10.9 |
| 4 | 11 | 20.0 |
| 5 | 24 | 43.6 |
| **Allocation concealment** |  |  |
| Adequate | 35 | 63.6 |
| Inadequate | 4 | 7.3 |
| Unclear / Not stated | 16 | 29.1 |
| **Type of analysis** |  |  |
| Intention to treat | 46 | 83.6 |
| As treated | 9 | 16.4 |
| **Funding source** |  |  |
| Pharmaceutical | 38 | 69.1 |
| Non pharmaceutical | 8 | 14.5 |
| Unclear / not stated | 9 | 16.4 |
| **Blinded adjudication of outcomes** |  |  |
| Yes | 47 | 85.5 |
| No | 5 | 9.1 |
| Not stated | 3 | 5.5 |

Supplementary References

1. Leyvraz PF, Richard J, Bachmann F, Van Melle G, Treyvaud JM, Livio JJ, Candardjis G: Adjusted versus fixed-dose subcutaneous heparin in the prevention of deep-vein thrombosis after total hip replacement. *N Eng J Med* 1983, 309:954-958.

2. Turpie AG, Levine MN, Hirsh J, Carter CJ, Jay RM, Powers PJ, Andrew M, Hull RD, Gent M: A randomized controlled trial of a low-molecular-weight heparin (enoxaparin) to prevent deep-vein thrombosis in patients undergoing elective hip surgery. *N Eng J Med* 1986, 315:925-929.

3. Planes A, Vochelle N, Mazas F, Mansat C, Zucman J, Landais A, Pascariello JC, Weill D, Butel J: Prevention of postoperative venous thrombosis: a randomized trial comparing unfractionated heparin with low molecular weight heparin in patients undergoing total hip replacement. *Thromb Haemost* 1988, 60:407-410.

4. Monreal M, Lafoz E, Navarro A, Granero X, Caja V, Caceres E, Salvador R, Ruiz J: A prospective double-blind trial of a low molecular weight heparin once daily compared with conventional low-dose heparin three times daily to prevent pulmonary embolism and venous thrombosis in patients with hip fracture.[see comment]. *J Trauma* 1989, 29:873-875.

5. Eriksson BI, Kalebo P, Anthymyr BA, Wadenvik H, Tengborn L, Risberg B: Prevention of deep-vein thrombosis and pulmonary embolism after total hip replacement. Comparison of low-molecular-weight heparin and unfractionated heparin. *J Bone Joint Surg Am* 1991, 73:484-493.

6. Lassen MR, Borris LC, Christiansen HM, Boll KL, Eiskjaer SP, Nielsen BW, Schott P, Olsen AD, Rodenberg JC, Lucht U: Prevention of thromboembolism in 190 hip arthroplasties. Comparison of LMW heparin and placebo. *Acta Orthop Scand* 1991, 62:33-38.

7. Levine MN, Hirsh J, Gent M, Turpie AG, Leclerc J, Powers PJ, Jay RM, Neemeh J: Prevention of deep vein thrombosis after elective hip surgery. A randomized trial comparing low molecular weight heparin with standard unfractionated heparin.[see comment]. *Ann Intern Med* 1991, 114:545-551.

8. Leyvraz PF, Bachmann F, Hoek J, Buller HR, Postel M, Samama M, Vandenbroek MD: Prevention of deep vein thrombosis after hip replacement: randomised comparison between unfractionated heparin and low molecular weight heparin. *Br Med J* 1991, 303:543-548.

9. Torholm C, Broeng L, Jorgensen PS, Bjerregaard P, Josephsen L, Jorgensen PK, Hagen K, Knudsen JB: Thromboprophylaxis by low-molecular-weight heparin in elective hip surgery. A placebo controlled study. *J Bone Joint Surg Br* 1991, 73:434-438.

10. The German Hip Arthroplasty Trial (GHAT) Group: Prevention of deep vein thrombosis with low molecular-weight heparin in patients undergoing total hip replacement. A randomized trial. *Arch Orthop Trauma Surg* 1992, 111:110-120.

11. Leclerc JR, Geerts WH, Desjardins L, Jobin F, Laroche F, Delorme F, Haviernick S, Atkinson S, Bourgouin J: Prevention of deep vein thrombosis after major knee surgery--a randomized, double-blind trial comparing a low molecular weight heparin fragment (enoxaparin) to placebo. *Thromb Haemost* 1992, 67:417-423.

12. Hull R, Raskob G, Pineo G, Rosenbloom D, Evans W, Mallory T, Anquist K, Smith F, Hughes G, Green D: A comparison of subcutaneous low-molecular-weight heparin with warfarin sodium for prophylaxis against deep-vein thrombosis after hip or knee implantation.[see comment]. *N Eng J Med* 1993, 329:1370-1376.

13. Platz A, Hoffmann R, Kohler A, Bischof T, Trentz O: Thromboembolieprophylaxe bei Hüftfraktur: Unfraktioniertes Heparin versus niedermolekulares Heparin (eine prospektive, randomisierte Untersuchung). *Z Unfallchir Versicherungsmed* 1993, 86:184-188.

14. Colwell CW, Jr., Spiro TE, Trowbridge AA, Morris BA, Kwaan HC, Blaha JD, Comerota AJ, Skoutakis VA: Use of enoxaparin, a low-molecular-weight heparin, and unfractionated heparin for the prevention of deep venous thrombosis after elective hip replacement. A clinical trial comparing efficacy and safety. Enoxaparin Clinical Trial Group. *J Bone Joint Surg Am* 1994, 76:3-14.

15. Fauno P, Suomalainen O, Rehnberg V, Hansen TB, Kroner K, Soimakallio S, Nielsen E: Prophylaxis for the prevention of venous thromboembolism after total knee arthroplasty. A comparison between unfractionated and low-molecular-weight heparin. *J Bone Joint Surg Am* 1994, 76:1814-1818.

16. Friedman RJ, Davidson BL, Heit J, Kessler C, Elliott CG, Cabanas V, Chenault C, Dall D, Chandler DR, Drennan D et al.: RD heparin compared with warfarin for prevention of venous thromboembolic disease following total hip or knee arthroplasty. *J Bone Joint Surg Am* 1994, 76:1174-1185.

17. Spiro TE, Johnson GJ, Christie MJ, Lyons RM, MacFarlane DE, Blasier RB, Tremaine MD: Efficacy and safety of enoxaparin to prevent deep venous thrombosis after hip replacement surgery. Enoxaparin Clinical Trial Group. *Ann Intern Med* 1994, 121:81-89.

18. Avikainen V, von Bonsdorff H, Partio E, Kaira P, Hakkinen S, Usenius JP, Kaaja R: Low molecular weight heparin (enoxaparin) compared with unfractionated heparin in prophylaxis of deep venous thrombosis and pulmonary embolism in patients undergoing hip replacement. *Ann Chir Gynaecol* 1995, 84:85-90.

19. Colwell CW, Spiro TE, Trowbridge AA, Stephens JW, Gardiner GA, Jr., Ritter MA: Efficacy and safety of enoxaparin versus unfractionated heparin for prevention of deep venous thrombosis after elective knee arthroplasty. Enoxaparin Clinical Trial Group. *Clin Orthop Relat Res* 1995, 321:19-27.

20. Hamulyak K, Lensing AW, Van der MJ, Smid WM, Van Ooy A, Hoek JA: Subcutaneous low-molecular weight heparin or oral anticoagulants for the prevention of deep-vein thrombosis in elective hip and knee replacement? Fraxiparine Oral Anticoagulant Study Group. *Thromb Haemost* 1995, 74:1428-1431.

21. Lee N, Rush J, Gilford E: Randomised trial of low molecular weight heparin versus warfarin in the prevention of venous thromboembolism after hip surgery [abstract] [abstract]. *J Bone Joint Surg Br* 1995, 77:60

22. Warwick D, Bannister GC, Glew D, Mitchelmore A, Thornton M, Peters TJ, Brookes S: Perioperative low-molecular-weight heparin. Is it effective and safe.[see comment]. *J Bone Joint Surg Br* 1995, 77:715-719.

23. Kalodiki EP, Hoppensteadt DA, Nicolaides AN, Fareed J, Gill K, Regan F, al Kutoubi A, Cunningham DA, Birch R, Harris N et al.: Deep venous thrombosis prophylaxis with low molecular weight heparin and elastic compression in patients having total hip replacement. A randomised controlled trial. *Int Angiol* 1996, 15:162-168.

24. Leclerc JR, Geerts WH, Desjardins L, Laflamme GH, L'Esperance B, Demers C, Kassis J, Cruickshank M, Whitman L, Delorme F: Prevention of venous thromboembolism after knee arthroplasty. A randomized, double-blind trial comparing enoxaparin with warfarin.[see comment]. *Ann Intern Med* 1996, 124:619-626.

25. Levine MN, Gent M, Hirsh J, Weitz J, Turpie AG, Powers P, Neemeh J, Willan A, Skingley P: Ardeparin (low-molecular-weight heparin) vs graduated compression stockings for the prevention of venous thromboembolism. A randomized trial in patients undergoing knee surgery. *Arch Intern Med* 1996, 156:851-856.

26. Schwartsmann CR, Cavalieri CR, Drumond SN, Maciel AC, Molina MAP, Garzella MM, Damin M: Estudo aberto, randomizado, comparativo, para avaliar a eficácia e segurança da enoxaparina comparada à heparina não fracionada na profilaxia o trombembolismo venoso em pacientes submetidos a artroplastia total do quadril. *Rev Bras Ortop* 1996, 31:797-808.

27. Francis CW, Pellegrini VD, Jr., Totterman S, Boyd AD, Jr., Marder VJ, Liebert KM, Stulberg BN, Ayers DC, Rosenberg A, Kessler C et al.: Prevention of deep-vein thrombosis after total hip arthroplasty. Comparison of warfarin and dalteparin. *J Bone Joint Surg Am* 1997, 79:1365-1372.

28. Heit JA, Berkowitz SD, Bona R, Cabanas V, Corson JD, Elliott CG, Lyons R: Efficacy and safety of low molecular weight heparin (ardeparin sodium) compared to warfarin for the prevention of venous thromboembolism after total knee replacement surgery: a double-blind, dose-ranging study. Ardeparin Arthroplasty Study Group. *Thromb Haemost* 1997, 77:32-38.

29. Yoo MC, Kang CS, Kim YH, Kim SK: A prospective randomized study on the use of nadroparin calcium in the prophylaxis of thromboembolism in Korean patients undergoing elective total hip replacement. *Int Orthop* 1997, 21:399-402.

30. Planes A, Vochelle N, Fagola M, Bellaud M, The Reviparin Study Group: Comparison of two low-molecular-weight heparins for the prevention of postoperative venous thromboembolism after elective hip surgery. *Blood Coagul Fibrinolysis* 1998, 9:499-505.

31. Adolf J, Fritsche HM, Haas S, Hennig FF, Horbach T, Kastl S, Koppenhagen K, Michaelis HC, Rhamanzadeh R, Summa W et al.: Comparison of 3,000 IU aXa of the low molecular weight heparin certoparin with 5,000 IU aXa in prevention of deep vein thrombosis after total hip replacement. German Thrombosis Study Group. *Int Angiol* 1999, 18:122-126.

32. Colwell CW, Jr., Collis DK, Paulson R, McCutchen JW, Bigler GT, Lutz S, Hardwick ME: Comparison of enoxaparin and warfarin for the prevention of venous thromboembolic disease after total hip arthroplasty. Evaluation during hospitalization and three months after discharge.[see comment]. *J Bone Joint Surg Am* 1999, 81:932-940.

33. Planes A, Samama MM, Lensing AW, Buller HR, Barre J, Vochelle N, Beau B: Prevention of deep vein thrombosis after hip replacement--comparison between two low-molecular heparins, tinzaparin and enoxaparin. *Thromb Haemost* 1999, 81:22-25.

34. The TIFDED Study Group: Thromboprophylaxis in hip fracture surgery: a pilot study comparing danaparoid, enoxaparin and dalteparin. *Haemostasis* 1999, 29:310-317.

35. Hull RD, Pineo GF, Francis C, Bergqvist D, Fellenius C, Soderberg K, Holmqvist A, Mant M, Dear R, Baylis B et al.: Low-molecular-weight heparin prophylaxis using dalteparin in close proximity to surgery vs warfarin in hip arthroplasty patients: a double-blind, randomized comparison. The North American Fragmin Trial Investigators.[see comment]. *Arch Intern Med* 2000, 160:2199-2207.

36. Kakkar VV, Howes J, Sharma V, Kadziola Z: A comparative double-blind, randomised trial of a new second generation LMWH (bemiparin) and UFH in the prevention of post-operative venous thromboembolism. The Bemiparin Assessment group. *Thromb Haemost* 2000, 83:523-529.

37. Lassen MR, Borris LC, Jensen HP, Poulsen KA, Ejstrud P, Andersen BS: Dose relation in the prevention of proximal vein thrombosis with a low molecular weight heparin (tinzaparin) in elective hip arthroplasty. *Clin Appl Thromb Hemost* 2000, 6:53-57.

38. Bauer KA, Eriksson BI, Lassen MR, Turpie AGG, the Steering Committee of the Pentasaccharide in Major Knee Surgery Study: Fondaparinux Compared with Enoxaparin for the Prevention of Venous Thromboembolism after Elective Major Knee Surgery. *N Eng J Med* 2001, 345:1305-1310.

39. Eriksson BI, Bauer KA, Lassen MR, Turpie AG, Steering Committee of the Pentasaccharide in Hip-Fracture Surgery Study: Fondaparinux compared with enoxaparin for the prevention of venous thromboembolism after hip-fracture surgery.[see comment]. *N Eng J Med* 2001, 345:1298-1304.

40. Fitzgerald RH, Jr., Spiro TE, Trowbridge AA, Gardiner GA, Jr., Whitsett TL, O'Connell MB, Ohar JA, Young TR, Enoxaparin Clinical Trial Group: Prevention of venous thromboembolic disease following primary total knee arthroplasty. A randomized, multicenter, open-label, parallel-group comparison of enoxaparin and warfarin.[see comment]. *J Bone Joint Surg Am* 2001, 83:900-906.

41. Haas S, Fareed J, Breyer HG, Victor N, Weber C, Bacher P, Reid U, Misselwitz F: Prevention of severe venous thromboembolism after hip and knee replacement surgery - A randomized comparison of low-molecular-weight heparin with unfractionated heparin [abstract]. *Blood* 2001, 98:707a

42. Heit JA, Colwell CW, Francis CW, Ginsberg JS, Berkowitz SD, Whipple J, Peters G, AstraZeneca Arthroplasty Study Group: Comparison of the oral direct thrombin inhibitor ximelagatran with enoxaparin as prophylaxis against venous thromboembolism after total knee replacement: a phase 2 dose-finding study. *Arch Intern Med* 2001, 161:2215-2221.

43. Turpie AG, Gallus AS, Hoek JA, Pentasaccharide I: A synthetic pentasaccharide for the prevention of deep-vein thrombosis after total hip replacement.[see comment]. *N Eng J Med* 2001, 344:619-625.

44. Eriksson BI, Bergqvist D, Kalebo P, Dahl OE, Lindbratt S, Bylock A, Frison L, Eriksson UG, Welin L, Gustafsson D et al.: Ximelagatran and melagatran compared with dalteparin for prevention of venous thromboembolism after total hip or knee replacement: the METHRO II randomised trial. *Lancet* 2002, 360:1441-1447.

45. Eriksson BI, Arfwidsson AC, Frison L, Eriksson UG, Bylock A, Kalebo P, Fager G, Gustafsson D: A dose-ranging study of the oral direct thrombin inhibitor, ximelagatran, and its subcutaneous form, melagatran, compared with dalteparin in the prophylaxis of thromboembolism after hip or knee replacement: METHRO I. MElagatran for THRombin inhibition in Orthopaedic surgery. *Thromb Haemost* 2002, 87:231-237.

46. Francis CW, Davidson BL, Berkowitz SD, Lotke PA, Ginsberg JS, Lieberman JR, Webster AK, Whipple JP, Peters GR, Colwell CW, Jr.: Ximelagatran versus warfarin for the prevention of venous thromboembolism after total knee arthroplasty. A randomized, double-blind trial. *Ann Intern Med* 2002, 137:648-655.

47. Lassen MR, Bauer KA, Eriksson BI, Turpie AG, European Pentasaccharide Elective Surgery Study (EPHESUS) Steering Committee: Postoperative fondaparinux versus preoperative enoxaparin for prevention of venous thromboembolism in elective hip-replacement surgery: a randomised double-blind comparison.[see comment]. *Lancet* 2002, 359:1715-1720.

48. Turpie AG, Bauer KA, Eriksson BI, Lassen MR, Study Steering Committee: Postoperative fondaparinux versus postoperative enoxaparin for prevention of venous thromboembolism after elective hip-replacement surgery: a randomised double-blind trial. *Lancet* 2002, 359:1721-1726.

49. Colwell CW, Jr., Berkowitz SD, Davidson BL, Lotke PA, Ginsberg JS, Lieberman JR, Neubauer J, McElhattan JL, Peters GR, Francis CW: Comparison of ximelagatran, an oral direct thrombin inhibitor, with enoxaparin for the prevention of venous thromboembolism following total hip replacement. A randomized, double-blind study. *J Thromb Haemost* 2003, 1:2119-2130.

50. Eriksson BI, Agnelli G, Cohen AT, Dahl OE, Lassen MR, Mouret P, Rosencher N, Kalebo P, Panfilov S, Eskilson C et al.: The direct thrombin inhibitor melagatran followed by oral ximelagatran compared with enoxaparin for the prevention of venous thromboembolism after total hip or knee replacement: the EXPRESS study.[see comment]. *J Thromb Haemost* 2003, 1:2490-2496.

51. Eriksson BI, Agnelli G, Cohen AT, Dahl OE, Mouret P, Rosencher N, Eskilson C, Nylander I, Frison L, Ogren M et al.: Direct thrombin inhibitor melagatran followed by oral ximelagatran in comparison with enoxaparin for prevention of venous thromboembolism after total hip or knee replacement. *Thromb Haemost* 2003, 89:288-296.

52. Francis CW, Berkowitz SD, Comp PC, Lieberman JR, Ginsberg JS, Paiement G, Peters GR, Roth AW, McElhattan J, Colwell CW, Jr. et al.: Comparison of ximelagatran with warfarin for the prevention of venous thromboembolism after total knee replacement. *N Eng J Med* 2003, 349:1703-1712.

53. Navarro-Quilis A, Castellet E, Rocha E, Paz-Jimenez J, Planes A, Bemiparin Study Group: Efficacy and safety of bemiparin compared with enoxaparin in the prevention of venous thromboembolism after total knee arthroplasty: a randomized, double-blind clinical trial.[see comment]. *J Thromb Haemost* 2003, 1:425-432.

54. Wang CJ, Wang JW, Weng LH, Hsu CC, Huang CC, Yu PC: Prevention of deep-vein thrombosis after total knee arthroplasty in Asian patients. Comparison of low-molecular-weight heparin and indomethacin. *J Bone Joint Surg Am* 2004, 86:136-140.

55. Colwell CW, Jr., Berkowitz SD, Lieberman JR, Comp PC, Ginsberg JS, Paiement G, McElhattan J, Roth AW, Francis CW, The EXULT BSG: Oral Direct Thrombin Inhibitor Ximelagatran Compared with Warfarin for the Prevention of Venous Thromboembolism After Total Knee Arthroplasty. *J Bone Joint Surg Am* 2005, 87:2169-2177.
